# Generating optimal control simulations of musculoskeletal movement using OpenSim and MATLAB

**DOI:** 10.7717/peerj.1638

**Published:** 2016-01-26

**Authors:** Leng-Feng Lee, Brian R. Umberger

**Affiliations:** Department of Kinesiology, University of Massachusetts Amherst, Amherst, MA, United States

**Keywords:** Predictive simulation, Dynamics, Musculoskeletal model, Optimization

## Abstract

Computer modeling, simulation and optimization are powerful tools that have seen increased use in biomechanics research. Dynamic optimizations can be categorized as either data-tracking or predictive problems. The data-tracking approach has been used extensively to address human movement problems of clinical relevance. The predictive approach also holds great promise, but has seen limited use in clinical applications. Enhanced software tools would facilitate the application of predictive musculoskeletal simulations to clinically-relevant research. The open-source software OpenSim provides tools for generating tracking simulations but not predictive simulations. However, OpenSim includes an extensive application programming interface that permits extending its capabilities with scripting languages such as MATLAB. In the work presented here, we combine the computational tools provided by MATLAB with the musculoskeletal modeling capabilities of OpenSim to create a framework for generating predictive simulations of musculoskeletal movement based on direct collocation optimal control techniques. In many cases, the direct collocation approach can be used to solve optimal control problems considerably faster than traditional shooting methods. Cyclical and discrete movement problems were solved using a simple 1 degree of freedom musculoskeletal model and a model of the human lower limb, respectively. The problems could be solved in reasonable amounts of time (several seconds to 1–2 hours) using the open-source IPOPT solver. The problems could also be solved using the fmincon solver that is included with MATLAB, but the computation times were excessively long for all but the smallest of problems. The performance advantage for IPOPT was derived primarily by exploiting sparsity in the constraints Jacobian. The framework presented here provides a powerful and flexible approach for generating optimal control simulations of musculoskeletal movement using OpenSim and MATLAB. This should allow researchers to more readily use predictive simulation as a tool to address clinical conditions that limit human mobility.

## Introduction

Dynamic models of the musculoskeletal system are powerful tools for studying the biomechanics of human movement. Musculoskeletal models are commonly used in conjunction with numerical optimization techniques to solve data-tracking or predictive human movement problems (Pandy, 2001; Umberger & Caldwell, 2014). In the tracking case, the objective is to minimize the difference between the behavior of the model and a target set of experimental data, such as joint kinematics and ground reaction forces (GRFs). In the predictive case, the objective is to perform the task while minimizing or maximizing a performance criterion, such as minimizing energy consumption or maximizing speed. The data-tracking approach has increasingly been used to address clinically-relevant human movement problems (e.g., Fey, Klute & Neptune, 2012; Goldberg, Ounpuu & Delp, 2003; Higginson et al., 2006). Predictive simulations of musculoskeletal motion likewise have many potential clinical applications, such as optimizing the design of assistive devices, predicting the outcomes of surgeries, and testing theories of movement control. The predictive approach is in many ways more powerful, given the ability to answer “what-if” types of questions, and the possibility to consider a wide range of conditions not limited to a set of experimental data. Despite these potential strengths, predictive musculoskeletal simulation has only seen limited use in clinical applications (e.g., Mansouri et al., 2016). This is due to many challenges such as the considerable computational demands (Anderson & Pandy, 2001), difficulty in defining relevant performance criteria (Ackermann & van den Bogert, 2010), and the substantial computer programming requirements involved.

Several commercial and open-source software packages are available that greatly facilitate modeling and simulation of the musculoskeletal system including OpenSim (Delp et al., 2007), AnyBody (Damsgaard et al., 2006), MSMS (Davoodi & Loeb, 2011) and SIMM/Dynamics Pipeline (Delp & Loan, 2000). In the present work, we utilized OpenSim because it is open-source and freely available, and it has a robust application programming interface (API). OpenSim provided a variety of tools for musculoskeletal modeling and simulation, such as for conducting forward dynamics and static optimization analyses. Among them, OpenSim provides a tool for generating tracking simulations without any programming required on the part of the user, employing an algorithm known as computed muscle control (Thelen & Anderson, 2006). However, the tools provided with the OpenSim end-user application do not provide the ability to generate predictive optimal control simulations. Users may extend the capabilities of OpenSim via the API, but this requires writing computer programs or plug-ins that interface directly with the OpenSim C++ libraries (Seth et al., 2011). Using this approach, it is possible for a knowledgeable programmer to write a C++ program to, for example, generate entirely predictive simulations of human walking (Dorn et al., 2015). Most of the C++ methods in recent versions of OpenSim (since version 3.0) are also accessible via scripting languages such as MATLAB (The MathWorks, Inc.) and Python (http://www.python.org). In this article, we focus on MATLAB due to its widespread use in the biomechanics community. MATLAB includes powerful design and control features and offers a more user-friendly programming environment than C++. Mansouri & Reinbolt (2012) recently linked OpenSim with MATLAB via the Simulink S-function API to create feedback controllers that act upon OpenSim models, allowing open- or closed-loop simulations to be run from within MATLAB. In the present work we employed a different approach, using the MATLAB scripting interface to the OpenSim API to solve musculoskeletal optimal control problems.

Optimal control is a general framework that has seen frequent use in solving musculoskeletal movement problems (Hatze, 1976; Davy & Audu, 1987; Pandy, Anderson & Hull, 1992). In the present work, we employed a direct collocation (DC) approach, which has been applied extensively in the aerospace field (Betts, 2010) and has recently seen increased use in biomechanics (e.g., Kaplan & Heegaard, 2001; Stelzer & von Stryk, 2006; Ackermann & van den Bogert, 2010; Kistemaker, Wong & Gribble, 2014). DC is well-suited for solving both predictive and tracking problems, as well as multi-objective problems that include weighted performance and tracking terms in the objective function (van den Bogert et al., 2012). In some cases, DC may hold a substantial performance advantage over traditional shooting methods. Ackermann & van den Bogert (2010) generated entirely predictive simulation of human walking with a two-dimensional (2-D) musculoskeletal model in about 30 min using DC on routine computer hardware. Our comparable simulations generated using a shooting method with a simulated annealing algorithm required over 48 hr when run on a high-performance computer workstation (Umberger, 2010). Another distinct advantage of the DC approach is that it can easily handle final-time equality constraints, such as the periodicity constraints that arise in simulating cyclical movements such as walking or running (van den Bogert, Blana & Heinrich, 2011).

With the DC approach, the original optimal control problem is converted to a parameter optimization problem by discretizing the states and controls on a temporal grid, and treating both the states and controls as unknowns in a general nonlinear programming (NLP) problem (Betts, 2010; Kaplan & Heegaard, 2001; van den Bogert, Blana & Heinrich, 2011). The MATLAB Optimization Toolbox includes a solver, fmincon, that can solve NLP problems with general equality, inequality and bound constraints. MATLAB can also interface with the open-source solver IPOPT (Wächter & Biegler, 2006) and the commercial solver SNOPT (Gill, Murray & Saunders, 2005) via the MEX-interface. IPOPT and SNOPT have the potential to substantially outperform fmincon by exploiting sparsity in the constraints Jacobian matrix that arises when the system dynamics are converted to a large set of algebraic equality constraints. In this paper, we focus on fmincon and IPOPT because fmincon is included with most installations of MATLAB and IPOPT is freely available. Moreover, we focus on predictive musculoskeletal simulation as OpenSim already provides the mean to generate tracking simulations via the computed muscle control algorithm.

In the work presented here, we combine the computational tools provided by MATLAB with the musculoskeletal modeling capabilities of OpenSim to create a framework for generating optimal control simulations of musculoskeletal movement using DC. This framework should allow biomechanics researchers to more easily and rapidly generate predictive simulations of human movement. We provide detailed results for a simple model with two antagonistic muscles, and we also evaluate the scalability of the approach on a larger 2-D model of the human lower limb. In order for other investigators to more easily apply this approach to their own research, we have made a complete working example freely available on the SimTK website (http://simtk.org/home/directcolloc).

## Materials and Methods

We begin by outlining the general optimal control problem formulation and then describe the way in which the capabilities of OpenSim and MATLAB were combined to solve these types of problems using the DC approach. Two examples are then presented to demonstrate the utility of the approach on a simple problem and on a larger-scale problem.

### Problem formulation

The optimal control problems presented herein can be stated as: find the states ***x***(*t*) and controls ***u***(*t*) that minimize an objective function

(1) \begin{eqnarray*}J = \int_0^T {L({\bi x}(t),{\bi u}(t))dt}\end{eqnarray*}

subject to constraints represented by the system dynamical equations

(2) \begin{eqnarray*}{\dot {\bi x}}(t) = {\bi f}({\bi x}(t),{\bi u}(t),t)\end{eqnarray*}

bound constraints on the states and controls

(3) \begin{eqnarray*}{{\bi x}_{min}} \le {\bi x}(t) \le {{\bi x}_{max}}\end{eqnarray*}

(4) \begin{eqnarray*}0 \le {\bi u}(t) \le 1\end{eqnarray*}

and problem-specific task constraints (Ackermann & van den Bogert, 2010; Davy & Audu, 1987; Pandy, Anderson & Hull, 1992). A common use of task constraints is to ensure periodicity of simulated cyclical motions, such as walking or running, by requiring that

(5) \begin{eqnarray*}{\bi x}(T) = {\bi x}(0)\end{eqnarray*}

(6) \begin{eqnarray*}{\bi u}(T) = {\bi u}(0)\end{eqnarray*}

where *T* is the final time. Additional or different task constraints may be specified for other specific movement problems, as will be seen in the examples presented here. The controls referred to above represent muscle excitations that were bounded between 0 (quiescent) and 1 (maximally excited) (Eq. 4). If desired, one can set the lower bound above 0 for part or all of the simulation time to require that a muscle be recruited above some threshold. Likewise, the upper bound can be set below 1 to prevent excitation above a prescribed, submaximal value.

The optimal control problems were converted to parameter optimization problems using the DC approach. The states and controls were discretized in time and the dynamical constraints (Eq. 2) were expressed as a large set of algebraic constraints using an Euler discretization

(7) \begin{eqnarray*}{{{x_{i + 1}} - {x_i}} \over {\Delta t}} - {{\bi f}_{i + 1}} = 0\end{eqnarray*}

where Δ*t* = *t*_*i* + 1_ − *t*_*i*_ and ***f***_*i* + 1_ represents the time derivatives of the state variables at time step *i*+1, as described in more detail in the next section. The reader is referred to the text by Betts (2010) and the appendix provided by Ackermann & van den Bogert (2010) for further details on the discretization scheme.

### OpenSim-MATLAB interface

**Figure 1:**
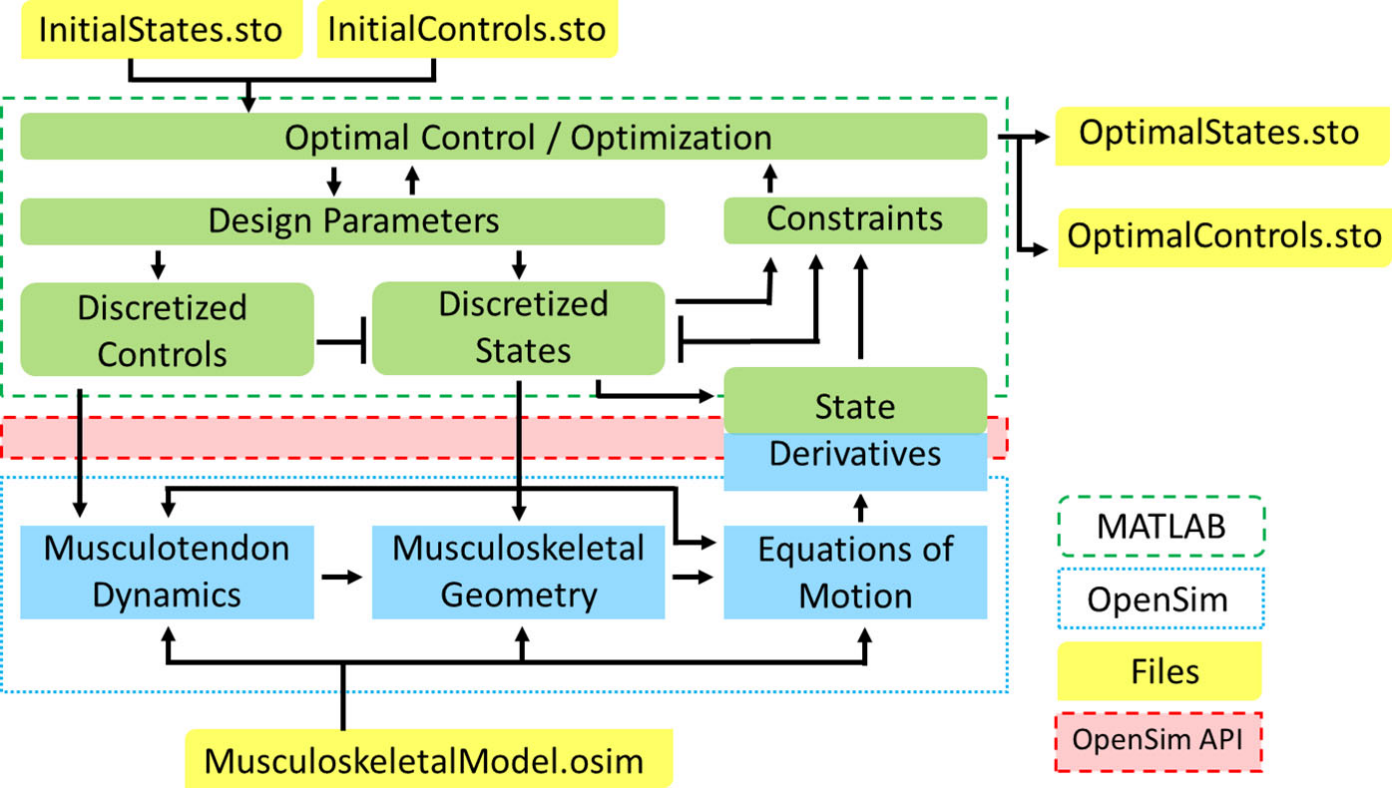
OpenSim-MATLAB interface for solving optimal control problems using direct collocation. Communication between MATLAB and OpenSim occurs via the OpenSim API. The green boxes represent the optimization process set up in MATLAB. The blue boxes represent the computational processes in OpenSim. The yellow boxes represent input and output files. The green-blue box labeled “State Derivatives” represents the discretization in the direct collocation approach. The initial guess and optimal result may be visualized in the OpenSim graphical user interface.

OpenSim was interfaced with MATLAB using the OpenSim API (Seth et al., 2011). MATLAB is used to set-up and solve the optimization problems and OpenSim is used to represent the dynamics of the musculoskeletal system (Fig. 1). OpenSim itself relies on the Simbody dynamics engine (not shown in Fig. 1) for multibody dynamics and other numerical operations (Sherman, Seth & Delp, 2011). The key link between OpenSim and MATLAB occurs at the block in Fig. 1 labeled “State Derivatives,” which corresponds to the Euler discretization scheme described by Eq. (7). The values of the vector term ***f***_*i* + 1_ in Eq. (7) may be obtained from OpenSim for a particular set of discretized states and controls by evaluating Eq. (2). If calculating the value of the objective function requires the magnitudes of any quantities that are implicit functions of the states and controls (e.g., contact forces, muscle powers), these may also be obtained by calling the appropriate OpenSim methods from within MATLAB.

The initial guesses for the optimization parameters are read from two OpenSim storage (.sto) files, labeled InitialStates.sto and InitialControls.sto in Fig. 1. The results of an optimization are written to two similar files, labeled OptimalStates.sto and OptimalControls.sto in Fig. 1. This allows the initial guesses and final results to be easily visualized in the OpenSim graphical user interface (GUI). Intermediate result files may optionally be written as an optimization progresses to allow the intermediate motion to be viewed in the OpenSim GUI. The storage files containing the final results allow for easy execution of forward dynamics simulations based on the DC results using the OpenSim ForwardDynamics tool. Forward simulations are generated using the states from the first time point in the OptimalStates.sto file as the initial conditions and the muscle excitations from all time points in the OptimalControls.sto files as the controls.

### Simple model

**Figure 2:**
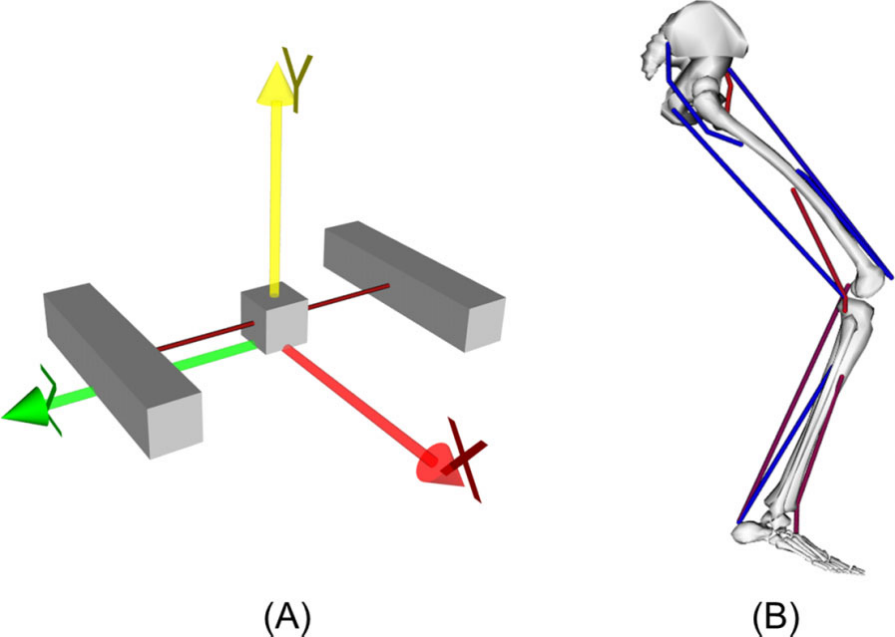
OpenSim models used in this project. (A) Simple model with 1 degree of freedom and two muscles. The central block can move freely along the Z-axis between the left and right anchor blocks. The simple model has 6 states and 2 controls, and was based on the Tug_Of_War.osim model provided with OpenSim. (B) Two-dimensional lower limb model with 3 degrees of freedom and 9 muscles. The lower limb model has 24 states and 9 controls, and was based on the leg6dof9musc.osim model provided with OpenSim.

To demonstrate the DC approach using the OpenSim-MATLAB interface, we used a simple 1 degree of freedom (DOF) model consisting of a block acted upon by two muscles, resulting in a model with 6 states and 2 controls (Fig. 2A). The model is able to translate along the mediolateral axis (Z axis in Fig. 2A) as it is acted upon by the two muscles, one of which pulls in the positive direction with the other one pulling in the negative direction. The simple model was modified from the “Tug of War” example (Tug_of_War.osim) that is provided with OpenSim. In the original example model, the block has 6 DOF and 5 constraints to produce uniaxial sliding. We replaced the 6 DOF free joint with a 1 DOF slider joint to eliminate the constraints and reduce the size of the state space. We also modified the tendon slack lengths from the example such that the muscles operate closer to the plateau of the force-length curve for the movement task that was simulated. For this study, both muscles had peak isometric forces of 1000 N, optimal fiber lengths of 0.25 m, tendon slack lengths of 0.05 m, and pennation angles of 0°.

Predictive simulations were generated where the target motion for the block was to begin at rest from a starting position of −0.08 m along the mediolateral axis, translate to a position of 0.08 m halfway through the movement time, and then return to the original state in a total movement time of 1.0 s. The actual movement was unspecified, other than for these task constraints defined at the initial time, the midpoint, and the final time. Other constraints were enforced such that the states and controls at the final time should match the states and controls at the initial time (Eqs. 5 and 6). The objective function was to minimize the sum of squared muscle activation integrals

(8) \begin{eqnarray*}J = {1 \over T}\sum\nolimits_{i = 1}^m {\int_0^T {a_i^2(t)dt} }\end{eqnarray*}

where *a_i_* is the instantaneous activation of the *i*th muscle and *m* is the number of muscles. The NLP problem was solved at a range of grid densities from 25–501 nodes (25, 51, 101, 151, 201, 301, 401 and 501 nodes). Solutions were obtained for IPOPT at all grid densities and for fmincon (interior-point algorithm) up to 201 nodes. The computation time for fmincon on the denser grids was too long (>1 day) to be of practical value for such a small problem.

An initial guess was generated by running a 1.0 s forward simulation where the model began static at an initial position of 0.0 m and did not move because the muscle controls were both set to zero. This will be referred to as the ‘static’ initial guess. For IPOPT, the NLP problem was solved two different ways; once using the static initial guess at all grid densities, and again using a grid refinement approach. For fmincon, the NLP problem was only solved using grid refinement, as convergence was too slow using the static initial guess. In the grid refinement approach, the initial guess at a particular grid density was the solution obtained from the next lower (i.e., coarser) grid density, except for the 25 node grid where there was no lower grid density. For example, the 101 node case was solved using the static initial guess and again using the optimal result obtained for the 51 node grid. For this particular movement task, we always used an odd number of nodes because of the constraint at the middle time point. For the 25 node case there were 200 unknowns and 156 constraints, while for the 501 node case there were 4008 unknowns and 3012 constraints. We evaluated the solutions by comparing the results obtained across the different grid densities, and by comparing the results at each grid density with forward simulations based on the optimal controls and optimal initial conditions obtained from the DC optimizations.

### Lower limb model

To evaluate the DC approach using OpenSim-MATLAB on a larger scale and more anatomically realistic model, we generated predictive simulations of lower limb movement using a sagittal plane, 3 DOF model of the human lower limb actuated by 9 muscles (Fig. 2B). The lower limb model had a total of 24 states and 9 controls. This model was modified from another example provided with OpenSim (leg6dof9musc.osim) and is based upon the standard three-dimensional OpenSim gait models (Delp et al., 1990; Anderson & Pandy, 2001) with a reduced set of muscles. The example model provided with OpenSim was modified to fix the pelvis segment in space and passive restraining moments were added to represent the contributions of ligaments and capsular tissues to the net joint moments (Davy & Audu, 1987).

For the lower limb model, predictive simulations were generated of a discrete, point-to-point movement. The model was required to move between an initial relaxed, hanging position and a final target posture (hip: flexed 80°, knee: flexed 85°, ankle: neutral 0°) in a fixed amount of time (0.75 s) while minimizing the sum of squared muscle activations (Eq. 8). At the final target posture, all generalized velocities had to be equal to zero. The desired initial and final states of the system were enforced using an appropriate set of task constraints. The motions and muscle activation patterns were unconstrained between the initial and final times. The initial guess for the lower limb optimizations was derived from a 0.75 s forward simulation where the joints were extended and the muscle controls were all set to an arbitrary, low value (2% of maximum). The lower limb movement problem was only solved using IPOPT, as convergence with fmincon proved too slow even on this modestly sized model. Results were obtained using grid refinement with grid densities of 25, 50, 100 and 200 nodes. The results at 100 and 200 nodes were nearly identical and detailed results are only presented for 200 nodes. For the 25 node case there were 825 unknowns and 615 constraints, while for the 200 node case there were 6600 unknowns and 4815 constraints. For this project, our goal with the lower limb model was to evaluate the feasibility of using the DC approach in OpenSim-MATLAB with a model more complex than the simple 1 DOF model, rather than analyzing the optimal motions and activation patterns and comparing them with actual human behaviors. For both the simple and lower limb models, the objective function gradient and the constraints Jacobian were approximated using forward finite differences. All optimizations were run on the same laptop computer with a 2.30 GHz Intel i5-5300U processor and 8 GB of RAM. The reported results were obtained using OpenSim release 3.3, MATLAB release 8.5, and IPOPT release 3.11.0.

## Results

**Figure 3:**
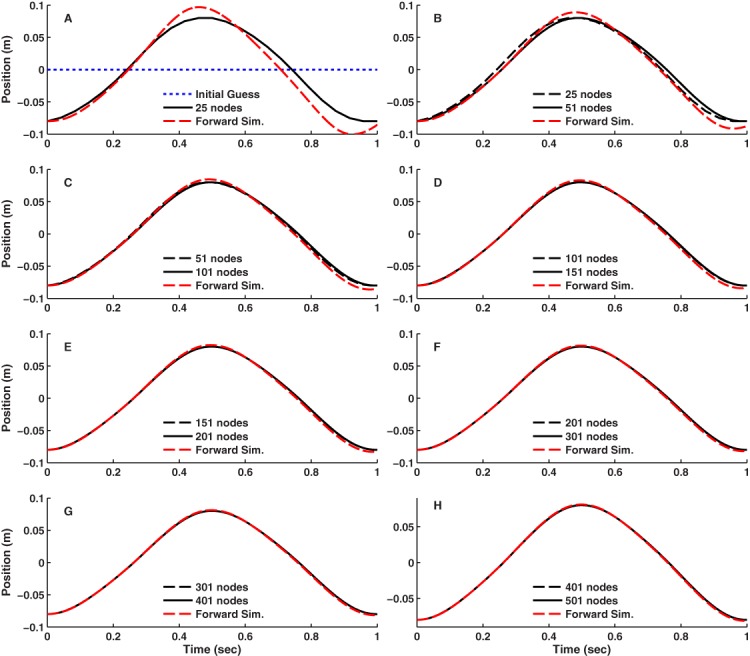
Position of the block versus time for the simple model optimal control problem. Result obtained using IPOPT are shown for different numbers of nodes using a grid refinement approach. Nearly identical results were obtained when the optimization at each node density was started from the static initial guess (blue dotted line in panel (A)). The results obtained using fmincon were also nearly identical to the IPOPT results up to 201 nodes. Solutions at greater node densities were not obtained using fmincon due to excessive computation time. Also shown in each panel are the results of a forward simulation (Forward Sim.) based on the optimal controls and initial conditions. The forward simulation results closely match the DC trajectories for node densities of 101 and greater.

**Figure 4:**
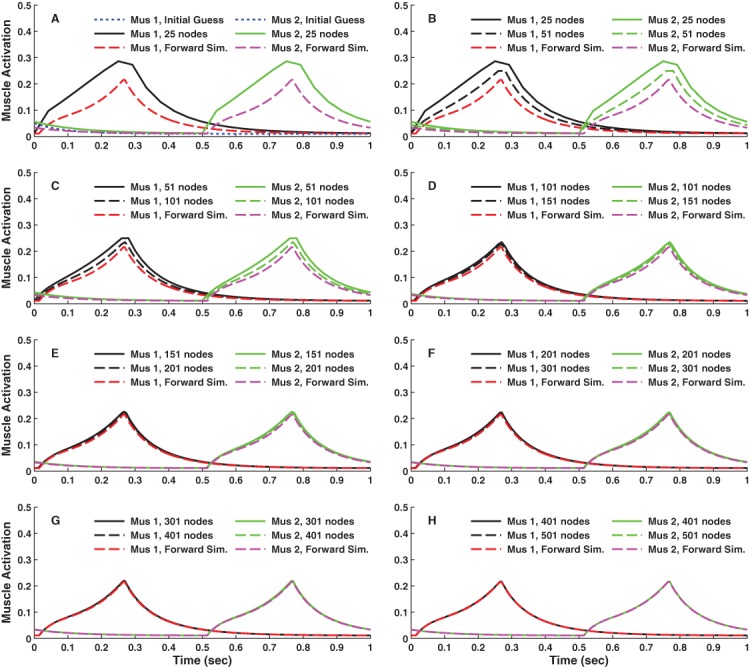
Muscle activations versus time for the simple model optimal control problem. Result obtained using IPOPT are shown for different numbers of nodes using a grid refinement approach. Nearly identical results were obtained when the optimization at each node density was started from the static initial guess (blue dotted line in panel (A)). The results obtained using fmincon were also nearly identical to the IPOPT results up to 201 nodes. Solutions at greater node densities were not obtained using fmincon due to excessive computation time. Also shown in each panel are the results of a forward simulation (Forward Sim.) based on the optimal controls and initial conditions. The forward simulation results closely match the DC trajectories for node densities of 151 and greater.

**Figure 5:**
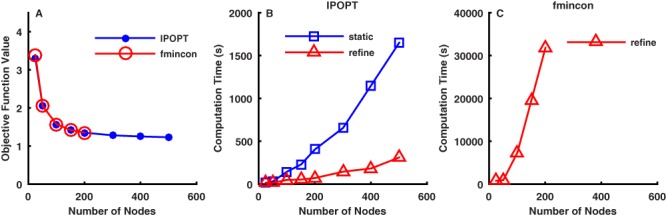
Optimization algorithm performance for the simple model optimal control problem. (A) Minimum objective function value (sum of squared muscle activation integrals, scaled by 100) for different numbers of nodes. At matched node densities, the IPOPT and fmincon solvers converged to the same object function values, except for a minor difference at 25 nodes. There was little difference in the minimum objective function value above 151 nodes. (B) Computation time using IPOPT with different numbers of nodes for the static initial guess (static) and using grid refinement (refine). The minimum objective function values were the same using the static initial guess and the grid refinement approach, but the results were obtained considerably faster using grid refinement. (C) Computation time using fmincon with different numbers of nodes for the grid refinement approach. Results were only obtained for fmincon using grid refinement up to 201 nodes. Convergence was too slow with fmincon using the static initial guess or using either approach above 201 nodes.

For the simple 1-DOF model, all node densities resulted in approximately sinusoidal motions (Fig. 3) with phasic muscle activity (Fig. 4) that satisfied the endpoint and midpoint constraints. This was true even when the initial guess (blue dotted lines in Figs. 3A and 4A) was far from the final result. There was little difference in the optimal motions above 101 nodes (Figs. 3D–3H) and little difference in the activations above 151 nodes (Figs. 4E–4H). Forward simulations based on the optimal controls and initial states reproduced the results obtained with DC for grid densities above 101 nodes. The results shown in Figs. 3 and 4 are for IPOPT using the grid refinement approach, where the initial guesses at each grid density were based on the optimal results obtained for the next lower grid density. The results were virtually identical when the static initial guess was used for all grid densities. However, it took on average 4 times longer for the optimizations to converge to the final solutions when starting from the static initial guess (Fig. 5B). The results for fmincon were also nearly identical to the IPOPT results using the grid refinement approach up to 201 nodes, which was the densest grid used with fmincon.

With increasing node density, the minimum objective function value (Fig. 5A), which is proportional to the area under the activation curves in Fig. 4, decreased considerably until 151 nodes, with little further reduction on denser grids. The same pattern was observed using either the static initial guess or grid refinement. For this particular problem, there would be little reason to use node densities greater than 201 nodes, as the results are nearly identical and the convergence time was substantially longer (Figs. 5B and 5C). Even at 25 nodes (Figs. 3A and 4A), the optimal results were qualitatively similar to the results obtained with greater numbers of nodes. The two solvers that were used, fmincon and IPOPT, generally converged to the same solutions; however, the execution times were dramatically different. Up to 201 nodes, fmincon took on average 260 times longer to converge that IPOPT (Figs. 5B and 5C).

**Figure 6:**
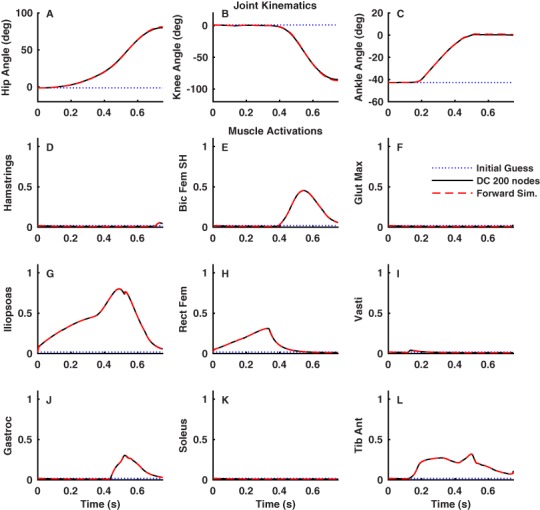
Joint kinematics (A–C) and muscle activations (D–L) for the lower limb optimal control problem. Result were obtained using IPOPT with a grid refinement approach (25, 50, 100 and 200 nodes). The dotted blue lines are the initial guess used at the 25 node density. The solid black lines are the optimal results for the 200 node density. The dashed red lines (overlying the solid black lines) are the results of a forward dynamics simulation (Forward Sim.) based on the optimal controls and initial conditions obtained at 200 nodes.

For the discrete, lower limb movement task, the model moved smoothly from the relaxed, initial state to the final, target posture in the specified amount of time (Fig. 6). The results were similar for all node densities and were nearly identical for 100 and 200 nodes (the 200 node results are shown in Fig. 6). Computation time was 1164 s at 25 nodes and 7802 s at 200 nodes. Forward simulations based on the optimal controls and initial states closely matched the DC results at 200 (Fig. 6) and 100 nodes, but did not match as closely at 50 and 25 nodes, consistent with the results obtained for the simple model. The optimal muscle activation patterns were consonant with the requirements of the simulated task and the minimum activation objective function. Activations were uniformly low in muscles that generate exclusively extension moments (Figs. 6F, 6I and 6K), while there were distinct bursts of activation in the muscles that generate only flexion moments (Figs. 6E, 6G and 6L). The results for biarticular muscles were more variable. The rectus femoris (Fig. 6H), which generates hip flexion and knee extension moments, was active until the knee joint started to flex around the middle of the movement time (Fig. 6B), at which point the gastrocnemius (Fig. 6J), which generates knee flexion and ankle extension moments, became active. When the rectus femoris activity ceased (Fig. 6H), iliopsoas activity increased (Fig. 6G), as it was the only remaining muscle that could generate the necessary hip flexion moment. The activity in the hamstrings (Fig. 6D), which generates hip extension and knee flexion moments, was low throughout the movement. All of the results for the simple and lower limb model optimal control problems are available on the SimTK website (http://simtk.org/home/directcolloc).

## Discussion

We used the MATLAB interface to the OpenSim API to develop a new framework for solving musculoskeletal optimal control problems. This approach effectively combines the high-level programming, design and control capabilities of MATLAB with the musculoskeletal modeling, simulation and analysis tools provided by OpenSim. Within this framework, we used the direct collocation technique to solve two predictive problems; a periodic motion problem using a simple musculoskeletal model and a discrete motion problem using a more realistic model of the human lower limb. Both problems were solved with reasonable computational demands using the IPOPT solver. The simple model optimal control problem could also be solved using the fmincon solver, but the computation times were too long to be of general use. Our intent is that this framework will facilitate the application of predictive biomechanical simulation to solving clinically-relevant human movement problems.

The IPOPT solver (Wächter & Biegler, 2006) greatly outperformed the fmincon solver from the MATLAB Optimization Toolbox (Figs. 5B and 5C). The performance advantage for IPOPT is primarily derived by exploiting sparsity in the constraints Jacobian matrix. Fewer than 5% of the elements of the constraints Jacobian were non-zero in the cases considered here, creating the opportunity for considerable computational efficiencies. However, IPOPT places more demands on the user, which can translate into considerable up-front costs. To use fmincon for the types of problems described here, the user needs to provide functions that return the value of the objective function and the values of the equality constraints. IPOPT has similar requirements, but also obligates the user to provide functions that return the gradient of the objective function with respect to the unknowns, the constraints Jacobian matrix, and the sparsity pattern of the constraints Jacobian. The fmincon algorithm will automatically calculate finite difference approximations for any derivatives that are not provided by the user; however, that is not the case for IPOPT. When using IPOPT with numerical derivatives, the user is responsible for issues such as choosing the ideal step sizes for the finite differences (Curtis & Reid, 1974) and calculating the non-zero elements of the sparse constraints Jacobian as efficiently as possible (Curtis, Powell & Reid, 1974). The process of determining the sparsity structure of the constraints Jacobian can itself be a time-consuming and error-prone task for problems with thousands of unknowns and constraint. However, that task need only be performed once for a particular model and movement problem, and the benefits can be substantial (compare times in Figs. 5B with 5C). The performance of both fmincon and IPOPT can benefit from analytical gradients and Jacobians if provided by the user, though IPOPT should still hold a considerable performance advantage due to the use of sparse linear algebra. Unfortunately, it is not always possible to obtain analytical expressions for the required derivatives when interfacing with OpenSim, which is a potential limitation of the approach presented here.

The objective function used in this work was an explicit function of the model states; therefore, it would be possible to derive an analytical expression for the gradient of the objective function with respect to the unknown parameters. However, the same is not true for the constraints Jacobian, which is where most of the time is spent in the optimization algorithms. OpenSim does not provide the full system dynamical equations in symbolic form, as could be obtained with dynamics software such as MotionGenesis (http://www.motiongenesis.com) or MapleSim (http://www.maplesoft.com/products/maplesim). OpenSim, via the Simbody dynamics engine, can return the time derivative of any state variable, or any other quantities of interest such as contact forces or muscle forces, but it does so without forming the relevant equations in full symbolic form (Sherman, Seth & Delp, 2011). An advantage of having the full symbolic equations is that analytical gradient vectors and Jacobian matrices can readily be determined, which should speed up the most time-consuming part of solving the NLP. There is a trade-off though, as the approach used by OpenSim has the advantage of greatly facilitating model development and analysis, while relieving the user from many lower-level details such as deriving symbolic equations of motions.

For the cases studied here, optimal results were obtained using IPOPT in times ranging from 15 s to 2 hr, depending on the node density and model complexity. The 2-D walking simulations generated by Ackermann & van den Bogert (2010) using a 50 node discretization had approximately the same number of unknowns and constraints as the lower limb movement simulations in the present study for the 100 node discretization. The computation time for the lower limb movement task for 100 nodes was 30 min, which is similar to the 35 min time reported for the 2-D walking simulations (Ackermann & van den Bogert, 2010). This comparison should be made cautiously as the computer used for the present work was likely faster, while Ackermann & van den Bogert (2010) used SNOPT, which has better convergence properties than IPOPT (van den Bogert, Blana & Heinrich, 2011). These two factors should at least partially offset, suggesting that this comparison may be reasonable as a first approximation. While the actual time required to generate walking simulations using IPOPT with the present framework will need to be determined, even if it requires several hours it will be highly competitive with traditional shooting methods (e.g., Anderson & Pandy, 2001; Miller et al., 2012; Neptune, Kautz & Zajac, 2001; Umberger, 2010). However, for tracking problems, the computed muscle control algorithm (Thelen & Anderson, 2006) that is included with OpenSim will likely be much faster than DC. Tracking problems involving large-scale, three-dimensional musculoskeletal models can be solved in a few hours or less using computed muscle control (Saul et al., 2015; Thelen & Anderson, 2006). Despite being slower, DC may still be preferred over computed muscle control for some tracking problems due to the flexibility it affords, such as in defining the cost function, or in placing arbitrary constraints on the solution. However, the actual computational demands of using DC via the OpenSim-MATLAB interface with large-scale musculoskeletal models will need to be evaluated in future research.

Convergence with the fmincon algorithm from the MATLAB Optimization Toolbox was too slow to be of much practical value, even for the simple model optimal control problem. This was due almost entirely to the inability of fmincon to make use of the known sparsity pattern of the constraints Jacobian. We informally compared the impact on performance of requiring IPOPT to use a dense Jacobian and found that it was only marginally faster than fmincon, rather than being over 100 times faster when the sparse Jacobian was used. Some of the other solvers in the MATLAB Optimization Toolbox (e.g., fsolve, lsqnonlin) can use sparsity information in the evaluation of Jacobian matrices, so perhaps future releases of fmincon will include this feature. Given access to a computer cluster, fmincon could also be run in parallel using the MATLAB Parallel Computing Toolbox. Given the large number of independent elements in the constraints Jacobian, performance could be dramatically increased given enough compute nodes. However, even without any performance enhancements, fmincon is still useful for development work as it is easier to use than IPOPT. We found that problems could be more easily tested and debugged using fmincon, before switching to IPOPT to gain the performance advantage.

A key aspect of the DC approach is deciding on the minimally acceptable grid density. For the lower limb discrete movement task, the 100 node solution was nearly indistinguishable from the 200 node solution, suggesting that the 100 node density could be used for future studies. However, this could only be determined by first solving the 200 node case. The cumulative computation time for obtaining the 200 node solution, including the grid refinement process, was over 3 hours. However, once that process was complete, related optimization problems, such as different final postures or different movement times, could be solved at the 100 node density in about 20–30 min each. Indeed, one of the strengths of DC is in rapidly solving several closely related optimization problems, once an initial problem has been solved (e.g., Ackermann & van den Bogert, 2010; van den Bogert et al., 2012). While we used fixed grid spacing in this work, it is possible to optimize the spacing used in the grid refinement process based on estimates of the discretization error at each grid density, which may confer additional performance benefits (Betts, 2010).

In this study, we leveraged the relatively new MATLAB interface to the OpenSim API. This allowed all of the programming to be done in the high-level MATLAB environment, while the musculoskeletal modeling and related numerical calculations were handled by the robust and efficient OpenSim C++ libraries. OpenSim itself relies on the Simbody dynamics engine (Sherman, Seth & Delp, 2011), which is built upon state-of-the-art numerical routines such as LAPACK (Anderson, 1999). Our use of MATLAB to interface with OpenSim is distinct from the approach reported previously where MATLAB was linked with OpenSim via a Simulink S-function (Mansouri & Reinbolt, 2012). In that project, an OpenSim model was wrapped in a Simulink block using the S-function API and then used to run both open-loop and closed-loop forward simulations from MATLAB/Simulink. That approach, and the one presented here, are indeed complimentary and simply suited to different purposes. While we used MATLAB in the current applications, the OpenSim API is also accessible from Python, as is the IPOPT solver. Python is an open-source high-level programming language with many numerical and scientific computing capabilities (Millman & Aivazis, 2011). Thus, it should be possible for other researchers to replicate the approach presented here using either MATLAB or Python.

In this project, we use DC to solve the optimal control problem (Kaplan & Heegaard, 2001; Ackermann & van den Bogert, 2010), but several other approaches have been used to generate simulations of a variety of human movements. The traditional approach has been to use a low-dimensional (e.g., Neptune, Kautz & Zajac, 2001; Umberger, 2010) or high-dimensional (e.g., Anderson & Pandy, 2001; Miller et al., 2012) discretization of the controls only, and then perform forward integrations of the dynamical equations in order to evaluate the objective function and constraints. Other recent approaches include modeling muscle reflexes (Geyer & Herr, 2010) and global parameterization of muscle forces using Fourier series (Shourijeh & McPhee, 2014). These other approaches could likely also be implemented using OpenSim and MATLAB and would be subject to many of the same strengths and weaknesses described herein. The example code provided with this article may prove to be a useful starting point for researchers implementing these other approaches via OpenSim and MATLAB.

## Conclusions

The OpenSim-MATLAB interface provides a powerful and flexible approach for generating optimal control simulations of musculoskeletal movement using the DC approach. This should facilitate the use of optimal control in developing therapies and assistive devices for clinical conditions that limit human mobility. Interested readers are encouraged to download and try the example provided on the SimTK website (http://simtk.org/home/directcolloc).

## Additional Information and Declarations

### Data Deposition

The following information was supplied regarding data availability: SimTK repository: http://simtk.org/home/directcolloc.
